# Metabolic Profiling Reveals an Abnormal Pattern of Serum Fatty Acids in MRL/lpr Mice Under Treatment With Prednisone

**DOI:** 10.3389/fphar.2020.00115

**Published:** 2020-02-25

**Authors:** Qianqian Li, Jia Zhou, Dingyi Zhang, Xiafeng Zhang, Zhenghao Xu, Dehong Wu

**Affiliations:** ^1^ The Second Affiliated Hospital of Zhejiang Chinese Medical University, Hangzhou, China; ^2^ School of Basic Medical Sciences, Zhejiang Chinese Medical University, Hangzhou, China

**Keywords:** systemic lupus erythematosus, prednisone, fatty acid, side effects, metabolic profiling

## Abstract

Glucocorticoids (GCs) are commonly used to treat systemic lupus erythematosus (SLE). Unfortunately, excessive GCs can induce many side effects associated with disordered fatty acid (FA) metabolism. Although an increased level of total FA has been found after GCs treatment, it is not clear whether all FA species increased or only certain FA species were altered. A gas chromatography–mass spectrometry-based FA profiling approach was performed to reveal the alterations of FA species in SLE model mice (MRL/lpr) after treatment with 5 mg/kg of prednisone. The study showed a distinct FA profile in MRL/lpr mice compared to the controls, mainly manifested by elevated polyunsaturated FAs (arachidonate, docosahexaenoate, *etc.*), which are related to the inflammatory state; and altered (product FA/precursor FA) ratios representing the estimated activities of FA desaturase and elongase (higher activities of multiple elongases, △4 desaturase, △5 desaturase, △6 desaturase, and lower activity of △8 desaturase). Treatment with 5 mg/kg of prednisone decreased the total level of n-6 polyunsaturated FA in MRL/lpr mice; in particular, the level of arachidonate and estimated activity of △5 desaturase were reduced to the control level. Moreover, prednisone induced additional perturbations in FAs, including not only saturated FAs, but also monounsaturated FAs and n-3 polyunsaturated FAs, indicating that there was a strong effect of prednisone on FA metabolism. These results may be valuable for further studies of the side effects of GCs treatment.

## Introduction

Systemic lupus erythematosus (SLE) is a typical, systemic autoimmune disease that causes damage to multiple organ systems (Lisnevskaia et al., 2014; Tsokos et al., 2016). Most SLE occurs in women of childbearing age, and environment, gene, infection and hormone factors are considered to contribute to the development of SLE. Although detailed research has been carried out, the pathogenesis of SLE is still not completely clear (Rekvig and Van der Vlag, 2014).

Epidemiological studies have shown that, compared with the general population, the prevalence of many metabolic disorders, such as abnormal glucose metabolism and disturbed lipid metabolism, has increased in patients with SLE (Tselios et al., 2016; Yan et al., 2016). Lipids are fundamental components of cells, and altered lipid metabolism can elevate the risk of cardiovascular disease and renal involvement in SLE patients (Frostegard et al., 2005; Teixeira and Tam, 2017). Fatty acid (FA) is an important kind of lipids with diverse functions, not only as part of cell membranes, but also as energy sources that can participate in a wide range of physiological and pathological conditions (Falomir-Lockhart et al., 2019). According to the saturation degree of hydrocarbon chains, FAs can be divided into three types: saturated fatty acids (SFAs), monounsaturated fatty acids (MUFAs) and polyunsaturated fatty acids (PUFAs).

Some species of FA can affect the inflammatory state of the body. For example, linoleic acid and arachidonic acid (AA) have pro-inflammatory effects (Alashmali et al., 2019), while docosahexaenoic acid (DHA) and eicosapentaenoic acid (EPA) have anti-inflammatory and immunomodulatory properties (Weylandt et al., 2012; Calder, 2015). Previous studies have found that abnormal FA levels are associated with insulin resistance and atherosclerosis (Lepretti et al., 2018; Steffen et al., 2018). Thus, it is speculated that the perturbations of FA metabolism may disrupt the metabolic homeostasis and immune balance of the body, and promote the development and progression of SLE complications. Currently, some researchers have shown altered FAs in patients with SLE (Aghdassi et al., 2011; Wu et al., 2012; Ormseth et al., 2013). However, due to the differences in disease activities and treatment strategies of patients, these studies have reached inconsistent conclusions.

Glucocorticoids (GCs) are widely used in the control and remission of SLE due to their effective anti-inflammatory and immunomodulatory effects. Recent studies have found that excessive GCs treatment can stimulate lipolysis, leading to an increase in the total level of FAs in the blood, which is one of the important factors of insulin resistance (Geer et al., 2014). Insulin resistance is not only associated with type 2 diabetes, but also with complications such as obesity and atherosclerosis (Bloomgarden, 2019). Although increased FA levels were found after GCs intervention, it was not clear whether all FA species increased or whether only certain FA species were altered. Therefore, a global analysis of FA profiles following treatment with GCs is necessary.

Metabolomics has the advantages of high throughout and high sensitivity, and is helpful to clarify the pathology of disease and the mechanism of drug action (Zhang et al., 2014; Luo et al., 2018; Tang et al., 2018). In this study, the FA metabolic profile of SLE model mice (MRL/lpr) was studied using the gas chromatography coupled with mass spectrometry (GC–MS) technique to reveal FA alterations related to SLE. In addition, the effects of prednisone (a kind of GCs) on FA metabolism were investigated.

## Materials and Methods

### Reagents

The 10% BF3/MeOH, the isotopically labeled internal standard (dodecanoic-12, 12, 12-d3 acid), sodium hydroxide (99.9%) and prednisone were purchased from Sigma-Aldrich (St. Louis, MO, USA). Methanol and n-hexane of HPLC grade were provided by Merck Chemicals (Darmstadt, Germany). Sodium chloride (99.9%) was purchased from Aladdin (Shanghai, China). A Milli-Q system (Millipore Corp, Millipore, MA, USA) was used to provide ultra-pure water.

### Animal Model and Drug Treatment

**Figure 1:**
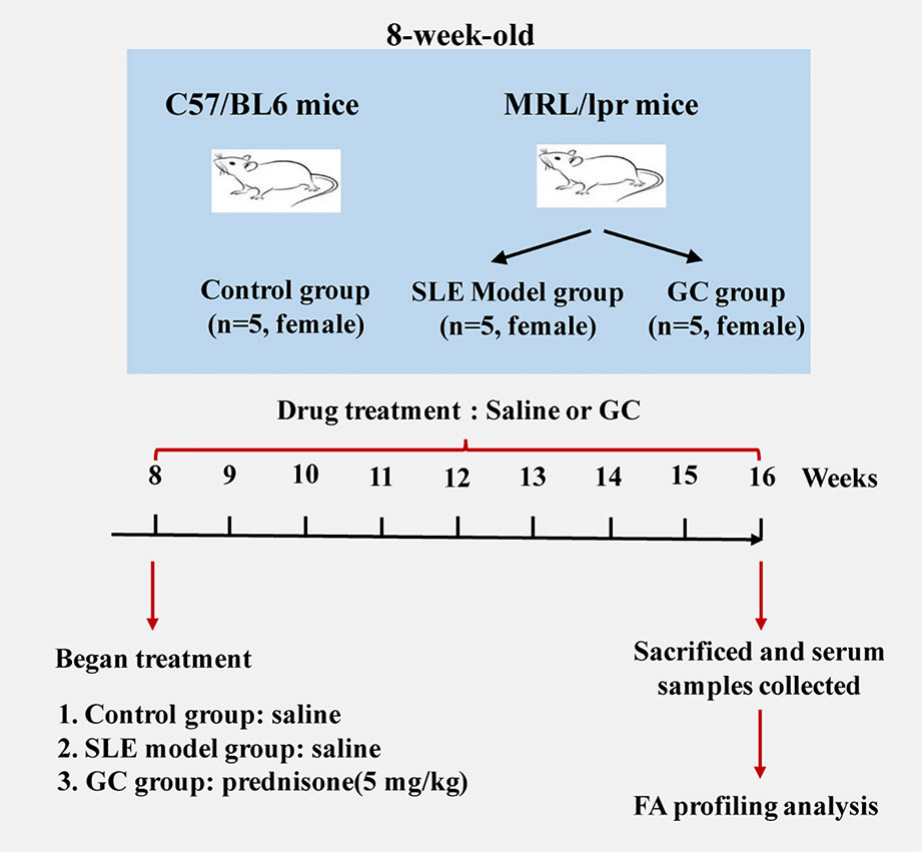
Schematic diagram of the study design (n = 5 for each group).

Eight-week-old female MRL/lpr (n=10) and C57/BL6 (n=5) mice were purchased from the Experimental Animal Center of Zhejiang Chinese Medical University (Hangzhou, China). The experimental protocol was approved by the Experimental Animal Health Ethics Committee of Zhejiang Chinese Medical University (license no: SCXK (Shanghai) 2012-0005). Experimental animals were maintained under specific pathogen-free conditions according to agency guidelines. Mice were kept with a 12-h light/dark cycle and with access to water and food *ad libitum*. Considering that SLE has a marked female predominance, we included only female mice in the experimental design, and five individual mice were used for each group. C57/BL6 mice served as the control group (n = 5). MRL/lpr mice were divided randomly into two groups: SLE model group (MRL/lpr group, n = 5) and GC group (n = 5). From the age of 8–16 weeks, mice in the GC group were intragastrically administered 5 mg/kg of prednisone suspension per day, which is equivalent to 0.5 mg/kg in humans. Model group and control group received an equal volume of saline accordingly. Mice were sacrificed 8 weeks after drug treatment, blood samples were collected from the eye socket vein and centrifuged at 845 × g for 10 min. The isolated serum samples were stored at –80°C. A scheme of the study design is shown in **Figure 1**.

### Sample Preparation for FA Profiling Analysis

A total of 20 μL of serum was added into a microcentrifuge tube containing 20 μL of internal standard solution (dodecanoic-12, 12, 12-d3 acid, 0.05 g/L). Subsequently, 200 μL of 0.5 mol/L NaOH/MeOH was added and mixed for 1 min. After incubation at 90°C for 5 min, 200 μL of 10% BF_3_/MeOH solution was added, and the mixture was incubated at 90°C for 5 min. After cooling, the sample was extracted using 200 μL n-hexane and 200 μL of saturated saltwater. The upper organic phase was concentrated and dried with a Labconco CentriVap system (Labconco, Kansas, MO, USA). Finally, 50 μL of n-hexane was added into the dried residue for reconstitution before GC–MS analysis.

### GC–MS Analysis

GC–MS analysis was performed on an Agilent 7890/5975C GC–MS (Agilent Technologies, Santa Clara, CA, USA). FAs were separated using a 30 m × 0.25 mm × 0.25 μm DB-5 MS fused silica capillary column (J&W Scientific, Folsom, CA, USA). The sample injection volume was 1 μL with a split ratio of 10:1. Helium (99.9996%) was used as the carrier gas and the flow rate was 1.2 mL/min. The injector and transfer line temperatures were both 280°C. The oven temperature program was: 60°C for 1 min, then ramped to 200°C at 20°C/min, and held for 3 min; later, ramped to 280°C at 5°C/min, and held for 5 min. The metabolic data were collected at the mass scan range from 50 to 500 amu after a solvent delay of 3.5 min. All samples were analyzed in a random order.

In order to monitor the repeatability of sample analysis, quality control (QC) samples were added into the analysis sequence for every 3 samples. QC samples were prepared by equally mixing the tested serum samples, and processed together with samples.

### Data Processing and Statistical Analysis

Serum FAs were identified and integrated by MSD ChemStation software (Agilent Technologies, Santa Clara, CA, USA). The peak area was normalized to the internal standard before further statistical analysis. Subsequently, the normalized data were subjected to a principal component analysis (PCA) using the SIMCA-P 11.0 version (Umetrics AB, Umea, Sweden) to display an overview of the difference of FA profiles among all groups. To further investigate the effects of different treatments (model or GC-treatment) on each FA, one-way analysis of variance (ANOVA) was performed with Tukey’s multiple comparisons test for *post hoc* comparison using SPSS 18.0 (International Business Machines Corp., Armonk, NY, USA). *P* values were adjusted using the Benjamini–Hochberg method. In addition, the ratio of product FA/precursor FA, an indicator of FA desaturase and elongase activity (Vessby et al., 2002; Warensjö et al., 2005), was calculated and compared between groups using ANOVA. Significantly changed FAs were clustered based on their Pearson correlation coefficients. All data were presented as mean ± SEM. Statistical significance was defined as *P* < 0.05, and a statistical trend was defined as *P* < 0.1.

## Results

### FA Metabolic Profiles of Mice Serum Samples

**Figure 2:**
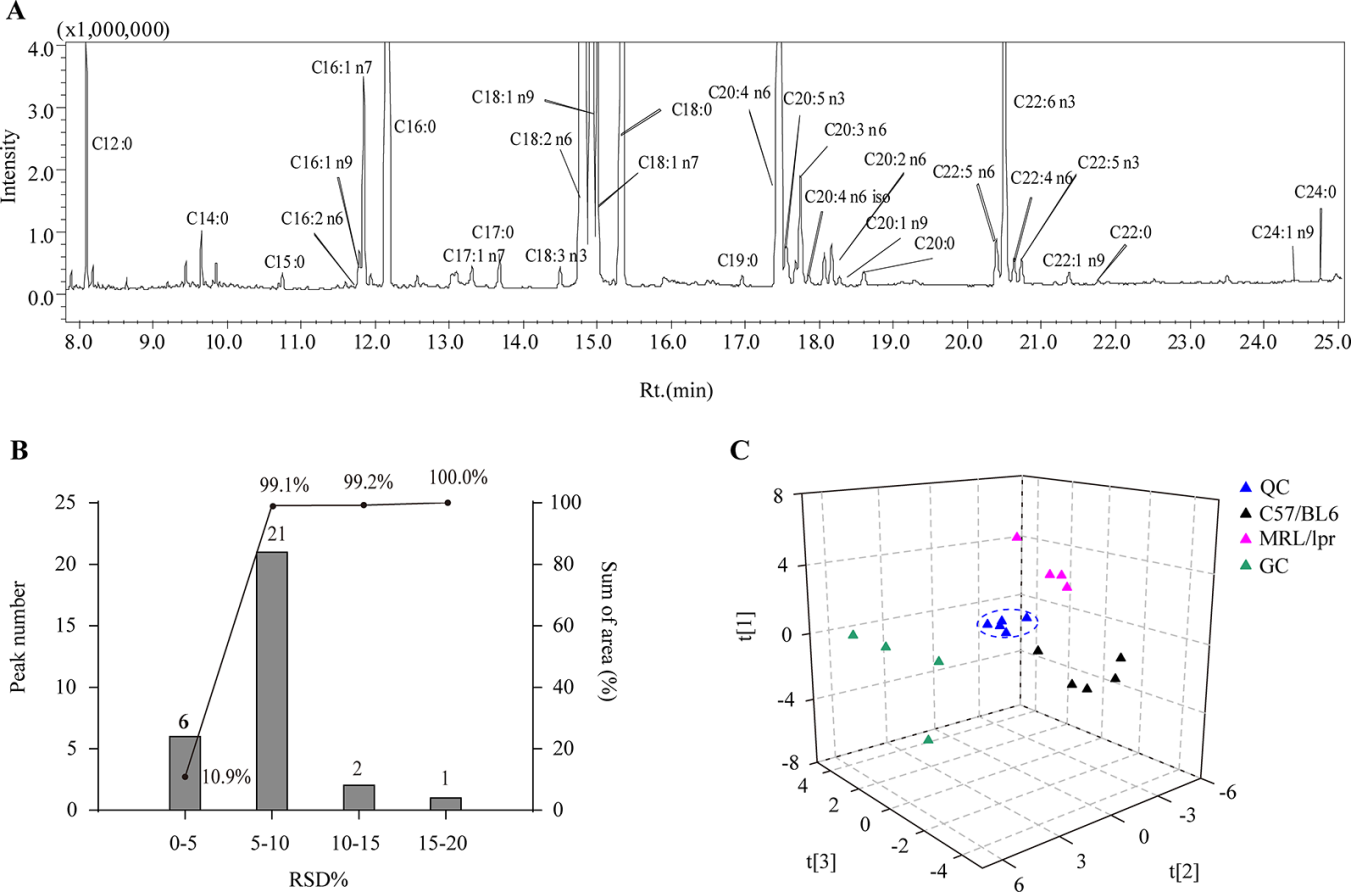
Analytical characteristics of the GC-MS-based FA profiling approach. **(A)** A representative total ion chromatogram of serum FAs. **(B)** RSD distribution plot of 30 species of FAs in five QCs; the columns indicate the number of FAs in the specified RSD range, and the line indicates the percentage of summed FA response in the specified RSD range. **(C)** PCA score plot of all samples based on FA profiles.

Thirty species of long chain FAs were detected in the serum samples (**Figure 2A**), including ten SFAs (C12:0, C14:0, C15:0, C16:0, C17:0, C18:0, C19:0 C20:0, C22:0 and C24:0), eight MUFAs (C16:1 n9, C16:1 n7, C17:1 n7, C18:1 n9, C18:1 n7, C20:1 n9, C22:1 n9, and C24:1 n9) and 12 PUFAs (C16:2 n6, C18:2 n6, C18:3 n3, C20:2 n6, C20:3 n6, C20:4 n6, C22:4 n6 iso, C20:5 n3, C22:4 n6, C22:5 n6, C22:5 n3, and C22:6 n3). The most abundant FAs included C12:0, C16:0, C18:0, C18:1 n7, C18:1 n9, C18:2, C22:5 n6, C20:5 n3, C18:2 n6, C22:6 n3, and C20:4 n6. After calculating the relative standard deviation (RSD) of each FA in QCs (n = 5), approximately 99.1% of the detected FAs showed an RSD below 10%, and all detected FAs had an RSD below 20% (**Figure 2B**), which demonstrated satisfactory robustness and reproducibility of the analytical method.

### Overview of the Differences in FA Profiles Among Groups

**Figure 3:**
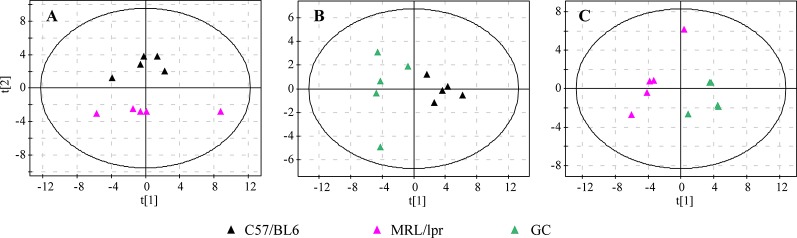
PCA score plots of different pairwise groups. **(A)** C57/BL6 and MRL/lpr groups, **(B)** C57/BL6 and GC groups, and **(C)** MRL/lpr and GC groups (n = 5 for each group).

In order to generally observe the differences of FA profiles in all samples, PCA was performed based on the serum FAs data from the control, model and GC groups. The 3D score plot showed a clear separation among the three groups, and QCs were clustered closely (**Figure 2C**). To further discriminate the effect of different treatments on FAs, PCAs of different pairwise groups were conducted. The distribution plots suggested that there was an obvious FA metabolic discrepancy between the control and MRL/lpr mice, and prednisone treatment caused significant perturbations in FA metabolism (**Figure 3**).

### Alterations in the Total Amount of FAs

**Figure 4:**
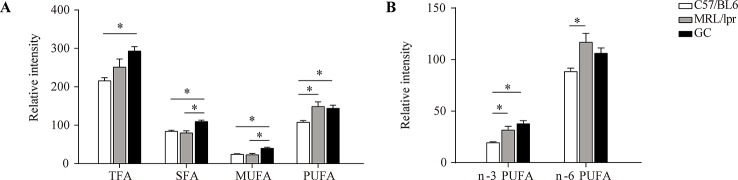
Changes in the total levels of various FAs. **(A)** TFA, SFA, MUFA and PUFA, and **(B)** n-3 PUFA and n-6 PUFA. Results are expressed as mean ± SEM (n = 5 for each group). The *P* values were obtained from ANOVA followed by Tukey’s *post hoc* test. *P < 0.05.

The total levels of SFA, MUFA, PUFA, and TFA (total fatty acid) in serum samples were calculated separately for each mouse. Compared to the control group, there were no significant changes in the total levels of SFA and MUFA in the MRL/lpr group, but the total PUFA level was significantly elevated, with both n-3 PUFA and n-6 PUFA increasing significantly (*P* < 0.05); the total levels of SFA, MUFA, and PUFA were all elevated in the GC group, while n-6 PUFA was returned to the control level. The total SFA and MUFA levels were significantly different between the model group and GC group (*P* < 0.05) (**Figure 4**).

### Changes of FA Species in MRL/lpr Mice

**Figure 5:**
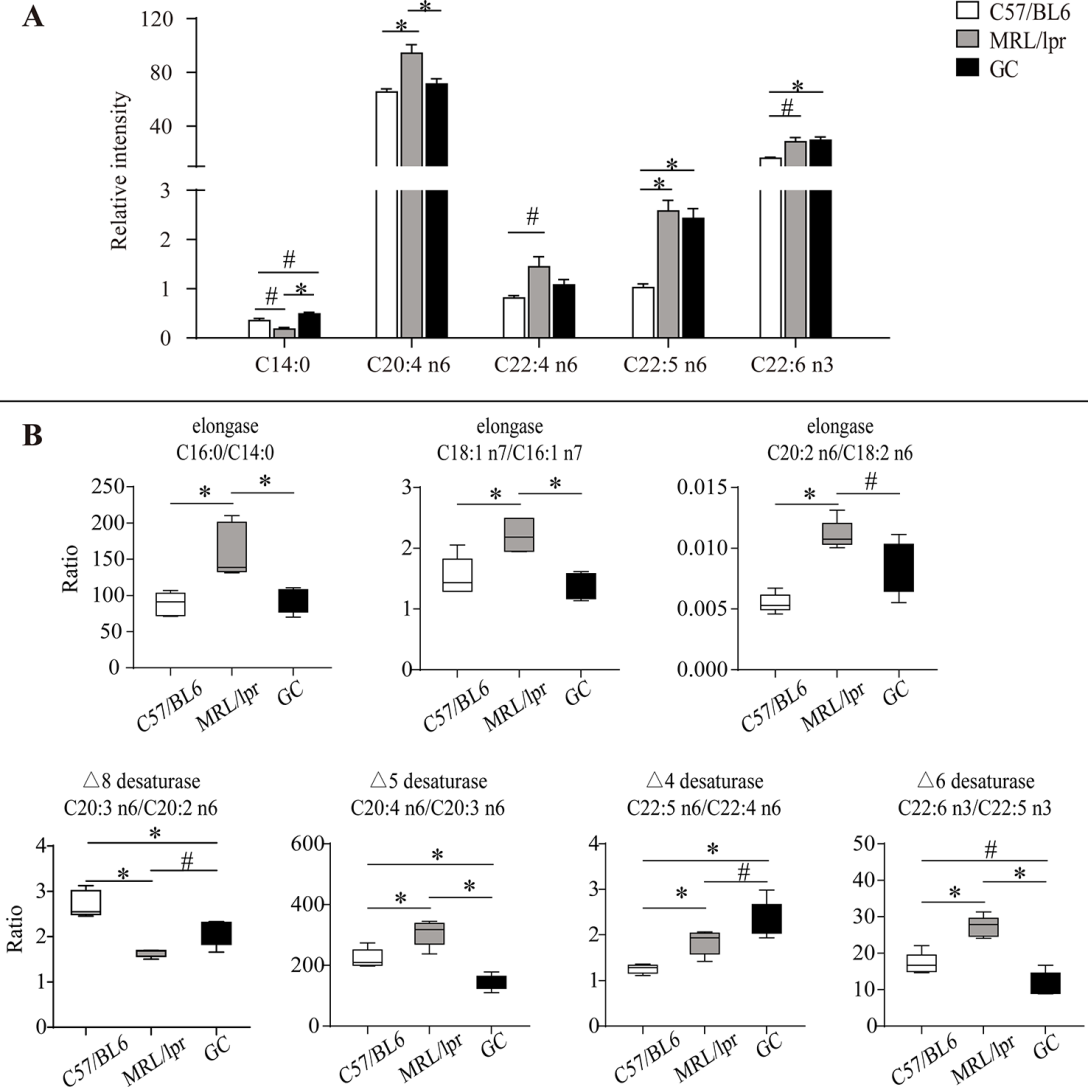
Alteration of serum FA species and product/precursor ratios in MRL/lpr mice compared to those in C57/BL6 mice. **(A)** Bar plots of changed FA species. Results are expressed as mean ± SEM (n = 5 for each group). **(B)** Box plots of changed product/precursor FA ratios (n = 5 for each group). The *P* values were obtained from ANOVA followed by Tukey’s *post hoc* test. *P < 0.05*, ^#^P* < 0.1.

To reveal the FA changes related to SLE, we measured the difference of each FA species between the control and model groups. The level of serum C14:0 was slightly decreased in MRL/lpr mice compared to the controls (*P* < 0.1). After the treatment with prednisone, the level of C14:0 was up-regulated and was a little higher than that of controls (*P* < 0.1) (**Figure 5A**).

Compared to the control group, some changes occurred in four kinds of serum PUFAs (C20:4 n6, C22:4 n6, C22:5 n6, C22:6 n3) in MRL/lpr mice. Among them, the levels of C20:4 n6 and C22:5 n6 were significantly increased (*P* < 0.05); moreover, C22:4 n6 and C22:6 n3 showed an increasing trend (*P* < 0.1) in the model group. After treatment with prednisone, C20:4 n6 and C22:4 n6 were down-regulated to the levels of those of the control group, but no changes occurred in C22:5 n6 and C22:6 n3 (**Figure 5A**).

### Estimation of Desaturase and Elongase Activities

The synthesis of unsaturated FAs is involved in several metabolic enzymes, such as desaturase and elongase. Desaturase can introduce double bonds, and elongase can lengthen carbon chains by adding two carbon atoms. Product/precursor FA ratios are generally employed to estimate the activities of FA desaturase and elongase (Vessby et al., 2002; Nakamura and Nara, 2004; Warensjö et al., 2005). In this study, the serum product/precursor FA ratios were computed and compared. As compared to the control group, seven ratios were significantly changed in the MRL/lpr group.

Three of these ratios were related to the estimated elongase activities, including (C16:0/C14:0), (C18:1 n7/C16:1 n7) and (C20:2 n6/C18:2 n6), which were significantly increased in MRL/lpr mice compared to those in C57/BL6 mice (*P* < 0.05) (**Figure 5B**). After treatment with prednisone, all three ratios were returned to the control group level.

The other four ratios were related to the estimated desaturase activities. Among them, (C20:4 n6/C20:3 n6), (C22:5 n6/C22:4 n6), and (C22:6 n3/C22:5 n3) were relatively higher in MRL/lpr mice than those in C57/BL6 mice, while (C20:3 n6/C20:2 n6) was decreased (*P* < 0.05). After treatment with prednisone, (C20:4 n6/C20:3 n6), and (C22:6 n3/C22:5 n3) were down-regulated, but (C20:3 n6/C20:2 n6) and (C22:5 n6/C22:4 n6) showed increases (**Figure 5B**).

### Additional FA Metabolic Perturbations After Treatment With Prednisone

**Figure 6:**
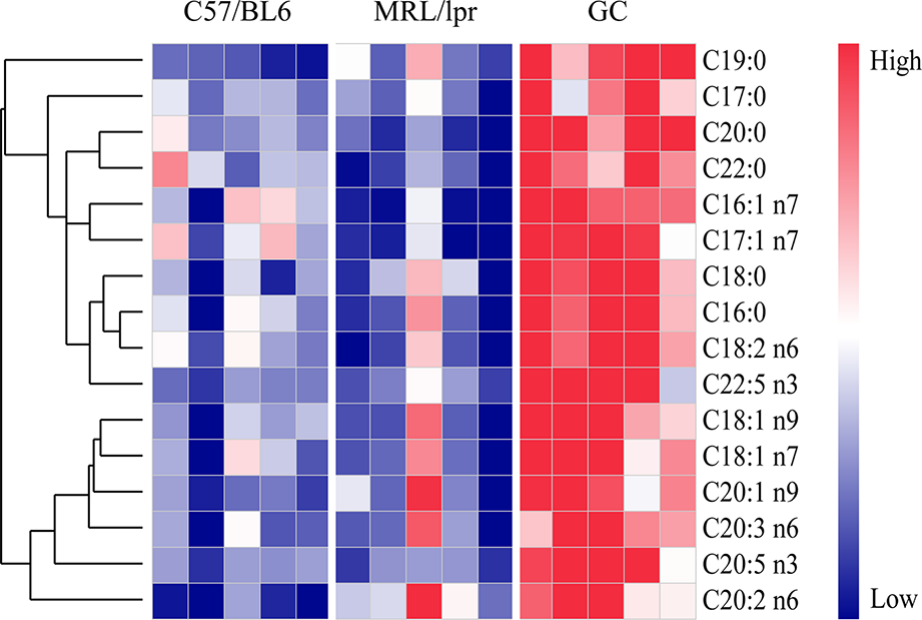
FA species with significant alterations under treatment with prednisone (n = 5 for each group). The FAs were clustered based on their Pearson correlation coefficients. Red represents higher concentrations, while blue represents lower concentrations.

Except for the SLE-related FAs, prednisone intervention caused additional changes in 16 species of FAs, including six species of SFAs (C16:0, C18:0, C20:0, C22:0, C17:0, and C19:0), five species of MUFAs (C16:1 n7, C17:1 n7, C18:1 n9, C18:1 n7, and C20:1 n9) and five species of PUFAs (C18:2 n6, C20:2 n6, C20:3 n6, C20:5 n3, and C22:5 n3) (*P* < 0.05) (**Figure 6**). All these FAs showed significant increases compared to the control and model groups, indicating a powerful effect of prednisone on serum FA metabolism.

## Discussion

**Figure 7:**
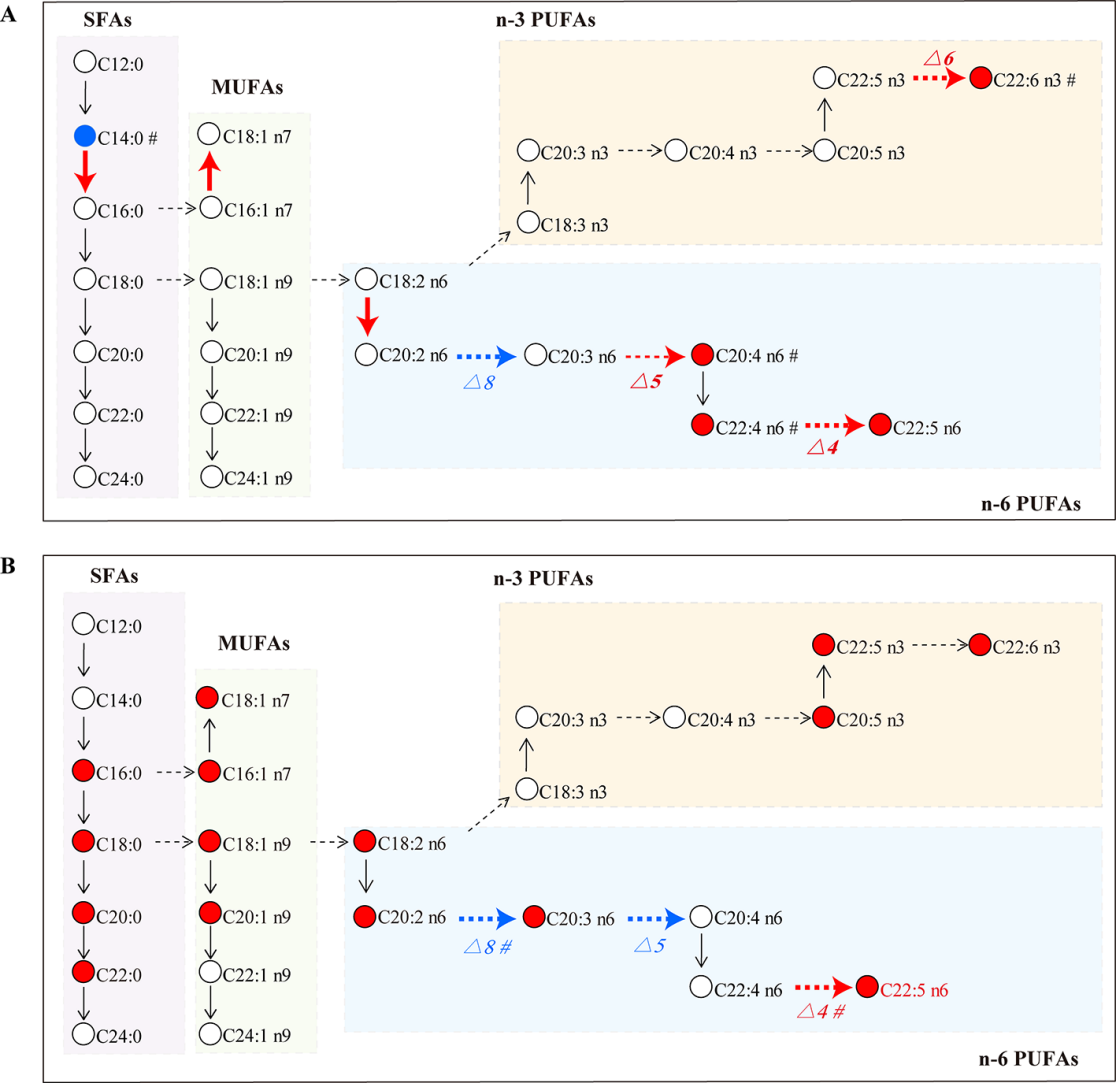
FA biosynthesis pathway changes in the MRL/lpr group **(A)** and the GC group **(B)** compared to the C57/BL6 group (n = 5 for each group). Dotted arrow: estimated desaturase activity; solid arrow: estimated elongase activity; red: significantly increased (*P* < 0.05); blue: significantly decreased (*P* < 0.05); #: slightly changed (*P* < 0.1).

SLE is an autoimmune inflammatory disease characterized by aberrant antibody response to multiple self-antigens and chronic inflammation that can involve multiple organs. GCs are commonly used to treat SLE, but superphysiological dosing can cause many side effects. Recent studies have found that GCs can lead to an increase in the total level of FAs in the blood, which is one of the important GC side effects (Arner, 2002). However, it was not clear which species of FAs were changed by GCs. Here, a GC–MS based FA profiling analysis was performed to comprehensively explore the specific FA changes related to SLE and FA metabolic perturbations induced by GCs. **Figure 7** summarizes the alterations of FAs and estimated desaturase/elongase activities in SLE model mice and GC-treated mice.

This study found that the total level of n-6 PUFA was elevated in MRL/lpr mice. Similarly, Aghdassi et al. (2011) observed an increase in n-6 PUFA in SLE patients. PUFAs are classified as n-3 and n-6 PUFAs according to the location of the double bonds. The derivates from n-3 PUFAs have anti-inflammatory and immunomodulatory properties (Weylandt et al., 2012; Calder, 2015), whereas those from n-6 PUFAs are generally known to have pro-inflammatory effects (Alashmali et al., 2019). Some specific n-6 PUFA changes related to SLE were discovered in this study. C20:4 n6 (arachidonic acid, AA) is a kind of n-6 PUFA, and we found that the level of AA was much higher in SLE model mice than in control mice. When stimulated by various factors, especially when an inflammatory reaction occurs, AA is released from the cell membrane, and oxidized by enzymes to produce eicosanoids (prostaglandin, thromboxane, leukotriene, etc.) (Calder, 2006), which is an important mediator of inflammation and is implicated in regulating the magnitude and persistence of inflammatory responses. Thus, we speculated that increased AA may promote the biosynthesis of pro-inflammatory eicosanoids and related pro-inflammatory cytokines, which is associated with the increased inflammation response in SLE model mice.

The level of C22:6 n-3 (docosahexaenoic acid, DHA) was increased in MRL/lpr mice. DHA is a potent inhibitor of NF-κB (nuclear factor κ B). The elevated levels of reactive oxygen species or other inflammatory signaling molecules (*e.g.* eicosanoid) can promote NF-κB activation and up-regulate the expression of many pro-inflammatory genes (IL-2, IL-6, IL-8, TNFα, *etc.*). DHA can directly or indirectly inhibit NF-κB by affecting G protein-coupled receptors and decreasing the release of phospholipase A2 to decrease the synthesis of eicosanoids, thus reducing the level of systemic inflammation (Calder, 2006). Thus, increased DHA may protect against inflammation in the SLE model mice. However, some studies of SLE patients get discrepant results, which may be related to the disease status, drug treatment and diet (Wu et al., 2012; Shin et al., 2017).

Long-chain FAs are mainly produced by enzyme-catalyzed palmitic acid extension and desaturation; the involved enzymes are elongase and desaturase, respectively. Elongase lengthens carbon chains by adding two carbon atoms, and desaturase introduces double bonds at specific positions on the carbon chain to increase the degree of unsaturation (Nakamura and Nara, 2004; Jakobsson et al., 2006). The activities of elongase and desaturase affect FA synthesis in the body, and the ratio of product/precursor FA in serum was used to evaluate the activities of FA desaturase and elongase (Yang et al., 2016). In this study, the ratios of (C16:0/C14:0), (C18:1 n7/C16:1 n7) and (C20:2 n6/C18:2 n6) were significantly increased in MRL/lpr mice compared to those in C57/BL6 mice, which suggested that the activity of corresponding elongase may be enhanced in model mice. (C20:4 n6/C20:3 n6) reflecting the activity of △5 desaturase, (C22:5 n6/C22:4 n6) reflecting the activity of △4 desaturase and (C22:6 n3/C22:5 n3) reflecting the activity of △6 desaturase were increased in MRL/lpr mice compared to those in C57/BL6 mice, while (C20:3 n6/C20:2 n6) reflecting the activity of △8 desaturase was decreased. MRL/lpr mice were found to have higher activities of △4 desaturase, △5 desaturase, △6 desaturase, and lower activity of △8 desaturase than control mice. The alteration in estimated desaturase activities might thereby affect the levels of downstream FAs (C22:6 n3, C20:4 n6, C22:4 n6 and C22:5 n6).

Prednisone is a widely used GC drug for anti-inflammatory and immunomodulatory therapies. Our results showed that the total level of n-6 PUFA was returned to the control level after treatment with prednisone. In particular, AA, an important precursor of pro-inflammatory eicosanoids, was down-regulated to the control level; moreover, the estimated activity of △5 desaturase (C20:4 n6/C20:3 n6 ratio) was reduced by prednisone, which may contribute to the decreased biosynthesis of AA. Inhibition of AA biosynthesis might be one of the anti-inflammatory mechanisms of prednisone. Previous researchers have found that GCs stimulate the decomposition of triacylglycerol to increase serum FA level by permitting and enhancing the lipolysis response to catecholamines, thyroxine and growth hormone, or acting on GC receptors of adipose cells (Xu et al., 2009). In our study, a wide range of FA metabolic perturbations were induced by prednisone, including the abnormal up-regulation of many species of SFAs, MUFAs and n-3 PUFAs, thus resulting in the elevation of the total FA level in blood. A persistent high level of FA is involved in the pathogenesis of insulin resistance (Arner, 2002), which may raise the risk of some complications in patients, such as type 2 diabetes, obesity, hypertension and coronary heart disease (Zappia and Monczor, 2019). Therefore, the perturbation effect of GCs on FA metabolism should be measured when using GCs to treat SLE, which could be an important indicator of the side effects of GCs.

## Conclusion

In this study, a GC–MS based FA profiling analysis was performed to reveal which species of FAs have been changed following disease and GCs treatment. The serum FA profile was altered in SLE model mice, mainly manifested by the increase of PUFAs, such as AA and DHA, which may be related to the inflammatory state. Moreover, some product/precursor FA ratios representing the estimated activities of desaturase and elongase were changed. Treatment with 5 mg/kg of prednisone reversed the change of n-6 PUFA, especially AA, which may be one of its anti-inflammatory mechanisms. Prednisone also induced a wide range of perturbations in FA metabolism; the FA level in blood was elevated, not just SFAs, but also MUFAs and n-3 PUFAs. These results may be valuable for further studies of the pathogenesis of SLE and the side effects of GC treatment.

## Data Availability Statement

All datasets generated for this study are available from the corresponding author on reasonable request.

## Ethics Statement

The animal study was reviewed and approved by the Experimental Animal Health Ethics Committee of Zhejiang Chinese Medical University.

## Author Contributions

Authors’ contributions were as follows: conception and design of the study (JZ, DW); animal experiment (DZ, XZ); metabolomics analysis (QL, DZ, XZ); data analysis and interpretation (JZ, ZX); drafting the article (QL, DW); and critical revisions for important intellectual content (JZ, DW). All authors read and approved the final manuscript.

## Funding

This study has been supported by the National Natural Science Foundation of China (No. 81403269, 81703966, 81873102), and Zhejiang Traditional Chinese Medicine Administration (No. 2017ZA064).

## Conflict of Interest

The authors declare that the research was conducted in the absence of any commercial or financial relationships that could be construed as a potential conflict of interest.
